# Orbital schwannoma with calcification treated by intracapsular excision: A case report

**DOI:** 10.1097/MD.0000000000024801

**Published:** 2021-02-19

**Authors:** Mingyu Ren, Yixiang Wu, Ruimiao Li, Jingjing Wang, Limin Liu, Yu Gao

**Affiliations:** Department of Orbital Disease and Ocular Tumor, Hebei Eye Hospital, Xingtai, Hebei Province, China.

**Keywords:** calcification, intracapsular excision, orbital schwannoma

## Abstract

**Rationale:**

Orbital schwannoma is a relatively rare orbital tumor, and calcification of the lesion is rarely found in the orbit. We report a case of orbital schwannoma which was characterized by calcification in the orbital muscle cone, and was cured by intracapsular excision.

**Patient concerns:**

A 54-year-old female with a complaint of a mass in the left orbit during a magnetic resonance imaging examination and symptom of dizziness 6 months before, presented with painless exophthalmos and vision decline in the left eye.

**Diagnoses:**

According to clinical manifestations, imaging examinations and postoperative immunohistochemical examinations, the diagnosis was orbital schwannoma, with calcification in the muscle cone.

**Interventions:**

The patient was treated by intracapsular excision of the left orbit. We removed the intracapsular mass and most part of the cyst wall in order to prevent orbital apex syndrome.

**Outcomes:**

The diagnosis of schwannoma with calcification was confirmed finally through histological and immunohistochemical exam. The patient was followed up for 28 months and the orbital CT scan showed that there were no significant lesions found in the orbital muscle cone.

**Lessons:**

Understanding clinical, imaging diagnostic, and histopathological features of rare orbital schwannoma with calcification will facilitate timely diagnosis and treatment of this condition. The intracapsular excision can help in avoiding complications.

## 1 Introduction

Orbital schwannoma is a relatively rare orbital tumor, accounting for 1% to 6% of all orbital tumors.^[1]^ Cyst formation is characteristic of orbital schwannomas.^[2]^ But calcification of the lesion is rarely found in the orbit. We report a case of orbital schwannoma which was characterized by calcification in the orbital muscle cone that was cured after intracapsular excision.

## 2 Case report

A 54-year-old east Asian female patient was found with a mass in the left orbit during a magnetic resonance examination performed 6 months ago for a complaint of dizziness. She was diagnosed with hypertension and cerebral infarction in the general hospital 6 months ago, and received systemic drug therapy. No special treatment was performed on her left orbit, and there was no significant change in ocular symptoms. Her systemic evaluation was unremarkable at the time of admission to our hospital. The patient presented with painless exophthalmos and vision decline in the left eye. There was no significant personal or familial medical history. Neurological signs were not found during physical examination. On ocular examination, the best-corrected visual acuity was 20/20 in the right eye and 20/200 in the left eye. The eye examination was unremarkable for eyelid, conjunctival, corneal, lenticular abnormalities, and fundus examination. Hertel exophthalmometry measured 12 mm in the right eye and 16 mm in the left eye. Intraocular pressure was 16 mmHg in the right eye and 18 mmHg in the left eye.

**Figure 1:**
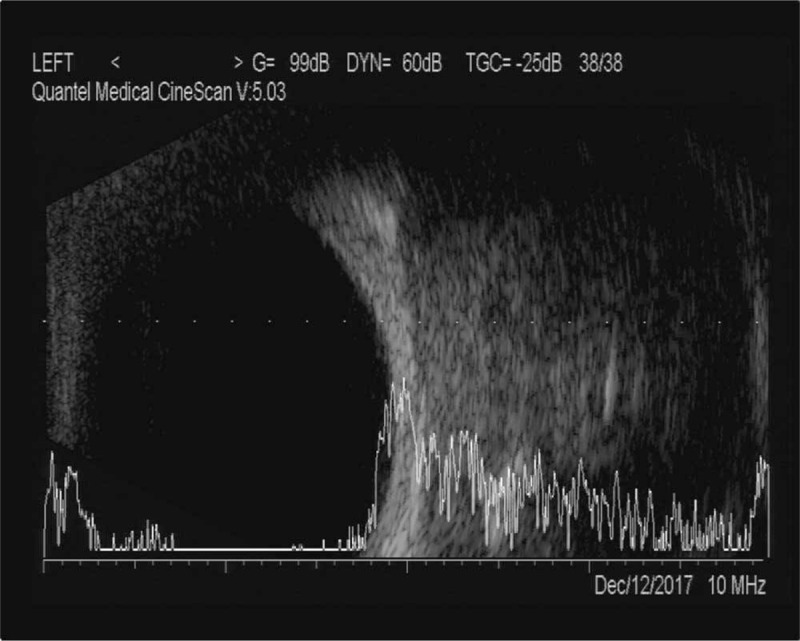
A/B-scan showed moderate echogenic lesions in the left eye. The echoes were dense, well-distributed, and the sound transmission was better. The patchy strong echoes and sound shadows were detected.

**Figure 2:**
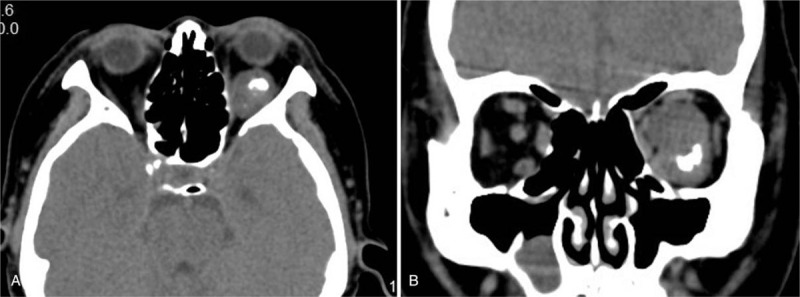
The images showed a well-defined mass in the left orbit muscle cone with a hyperdense speck suggestive of coarse calcification. (A) Axial CT scans. (B) Coronal CT scans.

**Figure 3:**
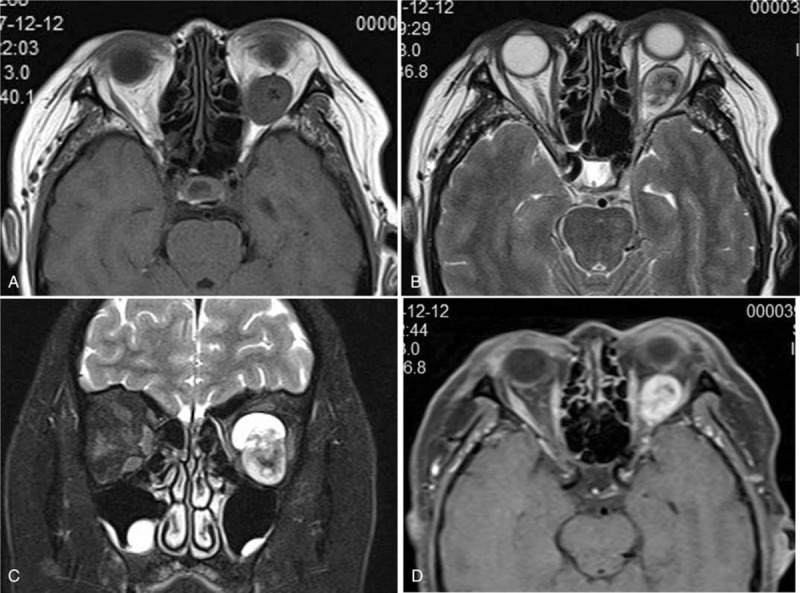
(A) The horizontal T_l_WI showed a well-defined tumor in the left orbital muscle cone, and irregular low signal in themedium signal lesion. (B) The horizontal T_2_WI showed high signal around the tumor and uneven low signal in the lesion. (C) The coronal scan showed the tumor in the left orbital muscle cone had uneven high signal with low signal in the lesion. (D) The scan showed the tumors can be unevenly enhanced.

A/B-scan showed moderate echogenic lesions in the left eye. The echoes were dense, well-distributed and the sound transmission was better. The patchy strong echoes and sound shadows were detected (Fig. 1). Orbital CT scan showed a well-defined soft tissue density mass in the left orbital muscle cone, with flaky high-density shadows seen within. The size of the mass was about 17 mm × 22 mm × 24 mm, with exophthalmos; extraocular muscles and optic nerve were compressed (Fig. 2). Orbital magnetic resonance imaging showed a circular-like mass in the left orbital muscle cone. T_1_-weighted images (T_1_WI) showed moderate signals, T_2_-weighted images (T_2_WI) were mixed signals, and most of them showed moderately high signals. Both T_1_WI and T_2_WI contained low-signal regions. Most part of lesion was significantly enhanced, while local lesions without enhancement (Fig. 3). The blood and urine tests were normal.

After preoperative examination, the patient underwent lateral orbitotomy approach to remove the left orbital mass under general anesthesia. During the operation, there was an oval tumor in the muscle cone of the left orbit. Its margins were well-defined, but it was very large, grey, unmovable, and adhesive to its surrounding tissues. The intracapsular excision was performed in this case. We just removed the intracapsular mass and most part of the cyst wall in order to prevent orbital apex syndrome. The surface of the tumor specimens was reddish whereas the interior was grey in color.

**Figure 4:**
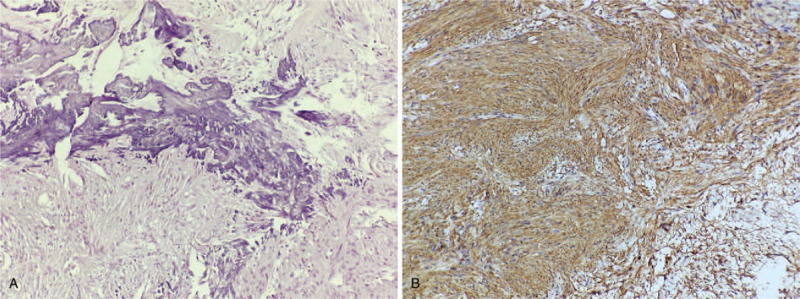
(A) The tumor was confirmed as schwannoma with calcification based on histological analysis (HE, ×200). (B) Immunohistochemical study revealed positive staining for S-100(S-100, ×200).

Histological examination revealed the tumor was schwannoma with calcification. Immunohistochemical study revealed positive staining for S-100, Vimentin, and Ki-67 (only 1%), while negative staining for NSE, Actin, and NF (Fig. 4).

**Figure 5:**
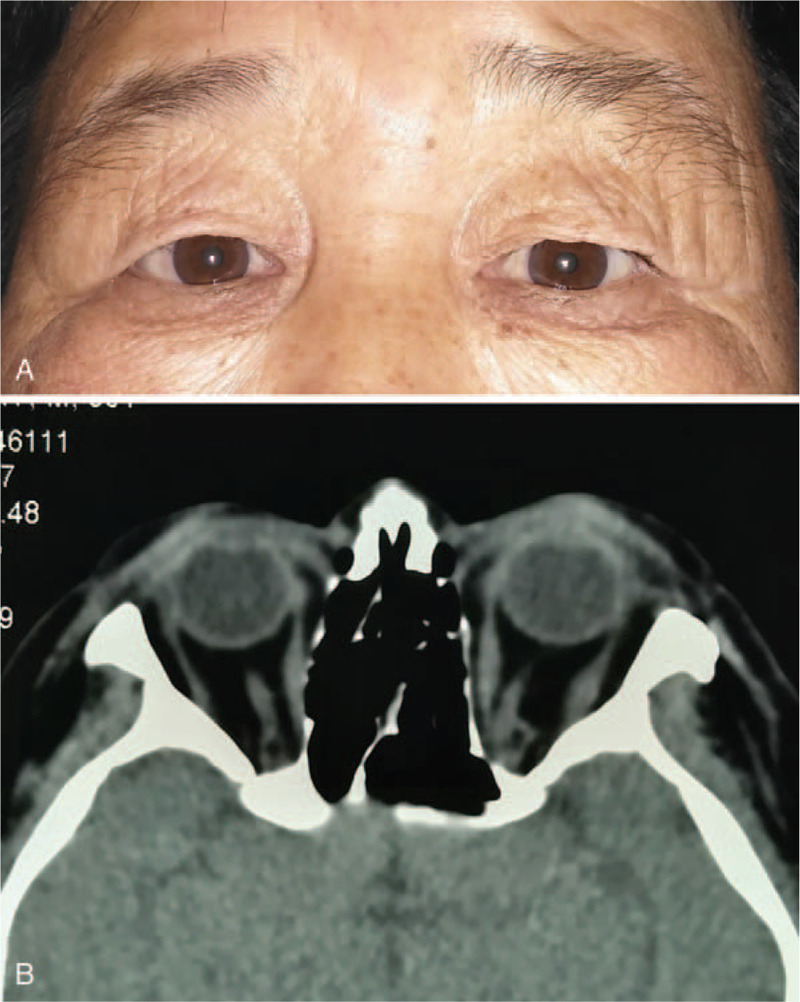
28 months after the surgery. (A) No abnormalities were observed in the patient. (B) The orbital CT scans showed the orbit was normal, and no significant lesions were observed.

The patient was followed up for 28 months. On ocular examination, the best-corrected visual acuity was 20/40 in the left eye; no abnormalities were observed in any direction of ocular movement. Hertel exophthalmometry measured 12 mm in the right eye and 11 mm in the left eye. The orbital CT scan showed that there were no significant lesions found in the orbital muscle cone (Fig. 5).

## 3 Discussion

Orbital schwannoma is usually a kind of benign neuroectodermal tumor, but occasionally malignant cases are encountered. Most of the tumors originate from branches of the oculomotor, trochlear, trigeminal, and abducens nerves, as well as from sympathetic and parasympathetic fibers. As a result, it is very difficult to identify the originating nerve clinically. Orbital schwannoma is usually asymptomatic during its initial stages,^[3]^ and it is mostly manifested as painless progressive lesions; its clinical manifestations are related to the location and size of the tumor. The common manifestations include exophthalmos and eyeball dislocation, and some patients may have the symptom of vision decline, diplopia, periorbital spontaneous pain, or tenderness.^[4]^

Orbital schwannoma is divided into Antoni type A and Antoni type B. Antoni type A is characterized by closely packed spindle cells having fusiform nuclei and eosinophilic cytoplasm. Antoni type B pattern is characterized by haphazardly distributed cells with distinct cytoplasmic margins.^[5]^ Secondary degenerative changes of the tumor, such as cyst formation, hyalinization, hemorrhage, are relatively common.^[6,7]^ However, calcification is extremely rare. Calcification results from the deposition of calcium salts in the body. From the pathological perspective, it can be divided into dystrophic calcification and metastatic calcification; the former is mainly caused by local microenvironmental changes, rarely accompanied by abnormal calcium metabolism; the latter is the calcium deposition caused by hypercalcemia, which is caused by metabolic disorder.^[8]^ In addition, the blood flow slows down or forms vortex, which causes thrombosis, calcium deposition, and formation of phleboliths. Venous hemangiomas and vascular malformations are common in this kind of diseases. The patient's systemic examination report showed normal, except cerebral infarction caused by hypertension, without any diseases related to abnormal calcium metabolism. Therefore, it is preliminarily speculated that the obvious calcification of the lesion may be caused by dystrophic calcification. The imaging examination showed that the tumor was located in the orbital muscle cone with less blood supply, and the lesion was large up to 17 mm × 22 mm × 24 mm. Due to relatively poor blood supply, calcification may have occurred.

When there is a lesion with calcification in the orbital muscle cone, orbital tumor such as the optic nerve tumors, vascular lesions, vascular malformations, other benign lesions and orbital malignancies should be taken into account.^[9]^ Phleboliths are mostly smooth and regular in shape, which often present in vascular lesions and vascular malformations. Irregular calcification is often present in malignant tumors and partially benign lesions. Calcification in schwannoma is rarely found in the orbit. In most typical cases, accurate preoperative diagnosis based on typical clinical and radiographic is usually available. However atypical presentations may pose a challenge.^[10]^ Definitive diagnosis of schwannoma is based on histopathological and immunohistochemical examinations of the surgical specimens.

The surgery to completely excise the intact tumor is the best goal for orbital schwannomas.^[11]^ Radiotherapy is an alternative treatment but is limited by unknown efficacy and collateral radiation damage.^[12]^ It is very important to take appropriate preoperative assessment of the tumors by orbital imaging, due to its variable presentation and location. In some cases, the lesions are in special locations, and the total resection can lead to serious complications. The lesion in this case was located in the orbital muscle cone, and the total resection may result in the vision loss, or the orbital apex syndrome, which will severely affect the quality of life. Moreover, the lesion was tightly adherent to its surrounding tissues, It is important to perform intracapsular resection, and remove the visible capsule well. When there are multiple recurrences and in cases where total excision is not possible, addition of topical Mitomycin-C may be an option to bring about tumor regression.^[12]^ However, it was not a best option in our case, because topical MMC may cause the optic nerve damage. In this case, there was no postoperative visual damage, and amazingly her visual acuity improved during follow-up. Moreover, long-term complications such as diplopia, eye movement disorders, ptosis were not detected. The recurrence of the lesion can occur after intracapsular excision. The cases reported in the literature describe late schwannoma recurrence at 1 months, 3, 6, and 22 years after initial excision. Incomplete surgical excision has been attributed to rapid recurrence. In addition, recurrence of orbital schwannoma is most likely associated with neurofibromatosis type 2 or schwannomatosis.^[12,13]^ Though the clinical follow-up in our patient showed improvement in the visual acuity and no muscle cone lesions in the orbital CT scan, we need to follow-up the patient for a long time period due to the possibility of recurrence.

## Author contributions

**Conceptualization:** Mingyu Ren, Yixiang Wu, Ruimiao Li.

**Data curation:** Ruimiao Li, Jingjing Wang, Limin Liu.

**Formal analysis:** Mingyu Ren, Yixiang Wu, Limin Liu.

**Investigation:** Yixiang Wu, Jingjing Wang, Yu Gao.

**Methodology:** Mingyu Ren, Yixiang Wu, Ruimiao Li.

**Project administration:** Mingyu Ren.

**Supervision:** Mingyu Ren, Yixiang Wu.

**Writing – original draft:** Mingyu Ren, Yixiang Wu, Ruimiao Li.

**Writing – review & editing:** Mingyu Ren, Yixiang Wu, Ruimiao Li, Limin Liu.
